# Comparative Analysis of *Ralstonia solanacearum* Methylomes

**DOI:** 10.3389/fpls.2017.00504

**Published:** 2017-04-13

**Authors:** Ivan Erill, Marina Puigvert, Ludovic Legrand, Rodrigo Guarischi-Sousa, Céline Vandecasteele, João C. Setubal, Stephane Genin, Alice Guidot, Marc Valls

**Affiliations:** ^1^Department of Biological Sciences, University of Maryland Baltimore CountyBaltimore, MD, USA; ^2^Center for Research in Agricultural Genomics, CSIC- IRTA- UAB -UBBarcelona, Spain; ^3^Department of Genetics, Universitat de BarcelonaBarcelona, Spain; ^4^Laboratoire des Interactions Plantes Micro-organismes, INRA, Centre National de la Recherche Scientifique, Université de ToulouseCastanet-Tolosan, France; ^5^Departamento de Bioquímica, Instituto de Química, Universidade de São PauloSão Paulo, Brazil; ^6^INRA, US 1426, GeT-PlaGe, GenotoulCastanet-Tolosan, France

**Keywords:** *Ralstonia*, methylome, comparative genomics, epigenomics, transcriptional regulation, transposon, nucleotide modification, genome

## Abstract

*Ralstonia solanacearum* is an important soil-borne plant pathogen with broad geographical distribution and the ability to cause wilt disease in many agriculturally important crops. Genome sequencing of multiple *R. solanacearum* strains has identified both unique and shared genetic traits influencing their evolution and ability to colonize plant hosts. Previous research has shown that DNA methylation can drive speciation and modulate virulence in bacteria, but the impact of epigenetic modifications on the diversification and pathogenesis of *R. solanacearum* is unknown. Sequencing of *R. solanacearum* strains GMI1000 and UY031 using Single Molecule Real-Time technology allowed us to perform a comparative analysis of *R. solanacearum* methylomes. Our analysis identified a novel methylation motif associated with a DNA methylase that is conserved in all complete *Ralstonia* spp. genomes and across the *Burkholderiaceae*, as well as a methylation motif associated to a phage-borne methylase unique to *R. solanacearum* UY031. Comparative analysis of the conserved methylation motif revealed that it is most prevalent in gene promoter regions, where it displays a high degree of conservation detectable through phylogenetic footprinting. Analysis of hyper- and hypo-methylated loci identified several genes involved in global and virulence regulatory functions whose expression may be modulated by DNA methylation. Analysis of genome-wide modification patterns identified a significant correlation between DNA modification and transposase genes in *R. solanacearum* UY031, driven by the presence of a high copy number of ISrso3 insertion sequences in this genome and pointing to a novel mechanism for regulation of transposition. These results set a firm foundation for experimental investigations into the role of DNA methylation in *R. solanacearum* evolution and its adaptation to different plants.

## Introduction

*Ralstonia solanacearum* is a widely-distributed, soil-borne phytopathogen belonging to the Betaproteobacteria subclass (Peeters et al., 2013). Known primarily as the causative agent of bacterial wilt among solanaceous plants, *R. solanacearum* encompasses a highly heterogeneous group of organisms capable of infecting over 200 plant species from more than 50 different families (Denny, 2007). Owing to its phylogenetic and host diversity, this group of organisms is conventionally known as the *R. solanacearum* species complex (RSSC) (Fegan and Prior, 2005). RSSC organisms share similar etiology, infecting and colonizing plant roots before invading xylem vessels and spreading to aerial plant parts. Extensive colonization of xylem vessels results in vascular dysfunction, leading to the signature wilting symptoms of *R. solanacearum* infections (Denny, 2007). Genomic analysis of sequenced *R. solanacearum* isolates has revealed that RSSC members share a similar genomic structure consisting of two circular replicons typically referred to as chromosome and megaplasmid (Remenant et al., 2010; Peeters et al., 2013). Multiple lines of evidence indicate that housekeeping genes reside predominantly in the chromosome, whereas environment- and pathogenicity-specific functions are encoded in the less-conserved megaplasmid (Genin and Denny, 2012). These include the main pathogenicity determinant of *R. solanacearum*, the type III secretion system (T3SS), as well as the extracellular polysaccharide (EPS) gene cluster and motility-associated determinants (Peeters et al., 2013). The notable phenotypic heterogeneity of *R. solanacearum* isolates has been primarily ascribed to the prevalence of genomic islands and genomic rearrangement events, frequently linked to the presence of prophages and transposable elements, as well as the ability of *R. solanacearum* to acquire exogenous DNA through natural transformation (Coupat et al., 2008; Remenant et al., 2010). Multi-locus sequence analyses, hybridization, genomic and phylogeographic methods have firmly established that the RSSC can be divided into four major phylotypes, further subdivided into sequevars and approximately corresponding to their known geographical origins (Guidot et al., 2007; Remenant et al., 2010; Wicker et al., 2012). However, the molecular mechanisms driving niche- and host-adaptation remain yet to be fully elucidated, prompting the need for novel approaches to understand their evolution.

DNA methylation is a chemical modification of DNA mediated by DNA methyltransferase (MTase) enzymes and known to directly regulate several processes in eukaryotic cells (Jones, 2012). DNA methylation is also prevalent in bacteria, in the form of 6-methyladenosine (m6A), 4-methylcytosine (m4C), and 5-methylcytosine (m5C) bases, and it is most frequently associated with the presence of restriction-modification (RM) systems. RM systems are composed of a restriction endonuclease (REase) and an MTase that preferentially bind to the same DNA sequence. They are broadly classified into four major types, according to their subunit composition, sequence recognition strategy, substrate specificity and cleavage position (Loenen et al., 2014). Methylation by MTases protects genomic DNA from cleavage and degradation by corresponding REases and, hence, RM systems are primarily envisaged as bacterial defense mechanisms against foreign DNA (Tock and Dryden, 2005). However, RM systems have also been shown to act as addiction molecules in plasmids and to help establish bacterial biotypes by preventing genetic exchange via conjugation or natural transformation (Handa and Kobayashi, 1999; Lindsay, 2010; Budroni et al., 2011). Furthermore, DNA methylation by RM systems and, more frequently, orphan MTases has been shown to be involved in coordinating replication initiation and cell-cycle progression, limiting transposition, regulating gene expression and phage packaging, and orchestrating phase-variation (Low and Casadesús, 2008).

The recent development of Single Molecule, Real-Time (SMRT) DNA sequencing allows detection of methylated bases in bacterial plasmids and chromosomes as characteristic delays in the real-time monitoring of the incorporation of nucleotides by individual DNA polymerase molecules (Schadt et al., 2013). For large DNA sequences, methylation motifs can be inferred as overrepresented patterns in the sequence context surrounding the modified base. Inferred motifs can then be matched to genome MTases on the basis of motif similarity to MTases with known specificity, via MTase subcloning or through resequencing of MTase mutants (Murray et al., 2012; Forde et al., 2015; Blow et al., 2016). The availability of SMRT sequencing has enabled the characterization of many new RM systems and their target motifs (Murray et al., 2012; Blow et al., 2016). It has also made it possible to identify additional phase-variation systems modulated by methylation (Blakeway et al., 2014), to identify RM systems that likely define clade boundaries (Nandi et al., 2015) and to trace evolutionary changes in MTase target recognition (Furuta et al., 2014). Here we used SMRT sequencing of the reference *R. solanacearum* GMI1000 strain (phylotype I, sequevar 18) and the highly-aggressive *R. solanacearum* UY031 strain (phylotype IIB, sequevar 1) to perform a comparative analysis of their DNA modification patterns. We identified the target motif of an m6A MTase conserved in both strains and across the *Burkholderiaceae*. Analysis of conserved methylation sites for this MTase revealed a clear enrichment in up- and downstream regions of coding sequences, and comparative analysis of their genetic context suggested that methylation targets are under strong purifying selection. Detection of hyper-methylated and non-methylated regions for this conserved m6A MTase identified several promoters where methylation could have a regulatory function. The modification profile of strain UY031 was found to correlate significantly with the presence of a multi-copy transposable element with a highly non-uniform modification pattern.

## Materials and methods

### Reference genomes

Twelve complete genomes of the *R. solanacearum* species complex available through the NCBI RefSeq service (RefSeq, RRID:SCR_003496) were used as a reference for comparative genomics analyses (Supplementary Table 1). In addition to the *R. solanacearum* GMI1000 (phylotype I, sequevar 18) and UY031 (phylotype IIB, sequevar 1) strains, these genomes include several phylotype IIB representatives (Po82, UW163, and IBSBF1503), a phylotype I (FQY-4), a phylotype III (CMR15) and a phylotype IV (PSI07) representative, as well as three additional *Ralstonia* species (*R. insidiosa, R. pickettii*, and *R. mannitolilytica*).

### Bacterial growth and genomic DNA preparation

Bacterial growth and genomic DNA extraction for the *R. solanacearum* UY031 strain was performed as described previously (Guarischi-Sousa et al., 2016). Briefly, *R. solanacearum* strain UY031 was grown in liquid rich B medium (10 g/l bactopeptone, 1 g/l yeast extract and 1 g/l casaminoacids) to stationary phase (OD_600 *nm*_ = 0.87). Genomic DNA was extracted from a bacterial culture grown to stationary phase to avoid overrepresentation of genomic sequences close to the origin of replication. Twelve ml of bacterial culture were used to extract DNA with the Blood and Cell Culture DNA Midi kit (QIAGEN, RRID:SCR_008539), following manufacturer's instructions for gram-negative bacteria. DNA concentration and quality were measured by spectrometry (Nanodrop 800; Thermo Fisher Scientific, RRID:SCR_013270). Bacterial growth and genomic DNA extraction for the *R. solanacearum* GMI1000 strain was performed in the present work. The protocol used to extract DNA from the GMI1000 strain was derived from the protocol described in Mayjonade et al. (2016). Briefly, bacteria were grown overnight in 50 ml MP minimal medium (FeSO_4_, 7H_2_O, 1.25 × 10^−4^ g/l; (NH_4_)_2_SO_4_, 0.5 g/l; MgSO_4_. 7H_2_O, 0.05 g/l; KH_2_PO_4_, 3.4 g/l) supplemented with glucose 20 mM and a pH adjusted to 6.5 with KOH. When the culture reached an OD_600 *nm*_ of 0.5 (exponential phase), bacteria were centrifuged 10 min at 7,000 rpm, the pellet was washed with 50 ml sterile water and centrifuged again to resuspend the pellet in 600 μl of lysis buffer (NaCl 2.5 M, TrisHCl pH8 1 M, EDTA pH8 0.5 M, SDS 20%, Sodium Metabisulfite 0.1%) preheated at 72°C. A total of 6 μl RNAse (100 mg/ml) was added before incubation 30 min at 55°C with gentle agitation every 10 min. Then 200 μl potassium acetate 5M was added, mixed and the suspension was centrifuged 10 min 13,000 rpm at 4°C. A total of 500 μl of supernatant was transferred in a new tube and 500 μl binding buffer (PEG8000 200 mg/ml, NaCl 200 mg/ml) was added. Then 30 μl of carboxylated magnetic beads (Thermo Fisher Scientific, RRID:SCR_013270) was added, and mixed before incubation for 1 h at room temperature under gentle agitation. The tubes were transferred to a magnetic rack to wash the beads 3 times with 70% Ethanol. DNA was eluted from the beads by resuspension in 80 μl of elution buffer (TrisHCl pH8 1M) preheated at 55°C. DNA concentration and quality were measured by spectrometry (Nanodrop 2000; Thermo Fisher Scientific, RRID:SCR_013270) and fluorometry (Qubit 3.0 Fluorometer; Thermo Fisher Scientific, RRID:SCR_013270). DNA integrity was evaluated by performing pulsed-field electrophoresis, which showed that the DNA molecules ranged in size from ~10 to ~90 kb with a mean at ~30 kb.

### SMRT sequencing

DNA libraries from strain UY031 were constructed using P5-C3 chemistry. The library preparation procedure followed the PacBio 128 standard for large insert library preparation with BluePippin size selection (Sage Science, 129 RRID:SCR_014808). The library insert size was 15 kb with size selection on the BluePippin using a 130 cut off of 6–50 kb for PacBioRSII. Whole-genome sequencing was performed using one single SMRTcell on PacBio RS II platform at Duke Center for Genomic and Computational Biology (USA). An assembly quality assessment was performed before all downstream analyses. All reads were mapped back to the assembled sequences using RS_Resequencing.1 protocol from SMRT Analysis 2.3. This analysis revealed that chromosome and megaplasmid sequences had 100% of bases called (percentage of assembled sequence with coverage > = 1) and 99.9999% and 99.9992%, respectively, of consensus concordance. More than 749 million of Pre-Filter Polymerase Read Bases were generated (>130x genome coverage) and deposited to NCBI Sequence Read Archive, RRID:SCR_004891 (SRP064191). Genomic DNA from the GMI1000 strain was sent to the Get-PlaGe core facility (INRA, Toulouse, France) where methylome data was obtained by SMRT technology. A 20-kb SMRTbell library was prepared according to manufacturer's protocols as described for the 20 kb template preparation with BluePippin size selections as follow: 5 μg of gDNA was sheared to an average length of 35 kb using Megaruptor system (Diagenode, RRID: SCR_014807), treated with DNA damage repair mix, end-repaired and ligated to hairpin adapters. Incompletely formed SMRTbell templates were digested using Exonuclease III and VII. Finally, the library was size selected with a 12 kb cutoff using BluePippin electrophoresis (Sage Science, RRID:SCR_014808). Sequencing was carried out on the PacBio RS II (INRA, Toulouse, France) from 0.25 nM of library loading on 3 SMRTCells, and using OneCellPerWell protocol on P6/C4 chemistry for 6 h movies, yielding mean genome coverage of 372x. All reads were mapped to the public GMI1000 reference genome using RS_Modification_and_Motif_analysis.1 protocol. This analysis revealed that both GMI1000 chromosome and megaplasmid sequences had 100% of bases called and 99.9952% and 99.9960%, respectively, of consensus concordance. 2.868.126.059 of Pre-Filter Polymerase Read Bases were generated (>450x genome coverage). Raw sequencing data was deposited on the NCBI Sequence Read Archive, RRID:SCR_004891 (SRP096275). Tet1-oxidation of DNA prior to SMRTbell library preparation, required for detection of m5C methylation (Clark et al., 2013), was not performed on either strain.

### Modification detection and motif analysis

The UY031 strain genome was assembled using RS_HGAP_Assembly.2 protocol from SMRT Analysis 2.3 (Chin et al., 2013) on one circular chromosome (3,412,138 bp) and one circular megaplasmid (1,999,545 bp). The origin of replication for both replicons was defined based on the putative origins of replication reported for reference strain GMI1000 (Salanoubat et al., 2002). The GMI1000 strain genome was assembled using the previously published GMI1000 genome as reference. Motif detection for both strains was performed using RS_Modification_and_Motif_analysis.1 protocol from SMRT Analysis using QV threshold of 30. The resulting modification files were deposited on the Gene Expression Omnibus (GEO) (GSE92982 and GSE93317; NCBI GEO DataSets, RRID:SCR_005012).

### Mapping of modification marks to genome features

Genome features were extracted from the NCBI RefSeq sequences of *R. solanacearum* GMI1000 (NC 003296.1, NC 003295.1) and *R. solanacearum* UY031 (NZ CP012688.1, NZ CP012687.1) using the BioPython 1.66 GenBank parser (Cock et al., 2009). A mapping between locus tag identifiers from the current RefSeq annotation and those from previous annotations was generated to facilitate identification of referenced genes in previously published work (Supplementary Table 2). For species reported in Blow et al. (2016), a Python script was used to identify and parse RefSeq sequences from GenBank identifiers, to download methylome General Feature Format (GFF) files from the corresponding GEO record (GSE69872; NCBI GEO DataSets, RRID:SCR_005012) and to associate methylome references in GFF files to RefSeq identifiers based on an exact match between the reported sequence length of the GFF reference and the RefSeq accession (Supplementary Table 3). For all species under analysis, modification marks were parsed from the corresponding GFF file using a custom Python script. Modification marks were then mapped to relevant genome features (CDS, tRNA, rRNA, tmRNA, ncRNA, mobile_element, and repeat_region) if their mark position overlapped the annotated feature positions. For coding features (CDS, tRNA, rRNA, tmRNA, and ncRNA), modification marks were annotated as *intragenic* if their positions mapped within the annotated coding segment, *upstream* if they mapped to the first non-coding 375 bp before the annotated feature start position, *downstream* if they mapped to the first non-coding 100 bp after the annotated feature end, and *intergenic* otherwise.

### Analysis of modification density

Modification density for a given type of modification mark was computed as the number of relevant modification marks within the region of interest divided by the length of said region. To account for correlation between sequencing coverage in a given region and its mark count, modification density within a given region was normalized with the ratio of genome-wide average coverage to region-wide average coverage for the mark type under analysis. Modification density plots were generated by analyzing normalized modification density using a sliding window of 1,000 bp with a step size of 100 bp.

### Analysis of conserved methylation marks

Conservation of detected methylation marks in the *R. solanacearum* GMI1000 and *R. solanacearum* UY031 genomes was assessed through alignment of their sequence context using a custom Python script. Bona fide orthologs between *R. solanacearum* GMI1000 and UY031 genes were obtained from a full-genome alignment with Mauve (Darling et al., 2004). For each ortholog pair, a pairwise gapless alignment was performed between the contexts of all modification marks mapping to the corresponding gene in either strain. Modification marks were labeled as conserved if their gapless context alignment had at least 70% identity and non-conserved otherwise. Modification marks not mapping to an ortholog pair were annotated as such. To assess modification mark conservation across the assembled panel of reference *Ralstonia* genomes, the sequence context of conserved modification marks in *R. solanacearum* GMI1000 was aligned with all reference genomes using BLASTN with modified gap penalties to avoid gapped alignments (Altschul et al., 1997). Modification marks were considered to be conserved in a particular reference species when the best BLASTN gapless alignment of their sequence context showed at least 70% identity. For each mark, the number of species against which valid alignments were obtained, the number of valid alignments with an intact 6 bp stretch in positions 17–22 (corresponding to the GTAW**A**C motif) and the number of alignments spanning the full mark context (41 bp) were compiled. For full alignments, the number of mismatches with respect to the *R. solanacearum* GMI1000 sequence in each alignment position was also computed.

### Identification of non-methylated, hyper-methylated and highly-conserved motifs

Non-methylated motif instances in the *R. solanacearum* GMI1000 and *R. solanacearum* UY031 genomes were identified following the protocol outlined in Blow et al. (2016). Essentially, a motif instance (detected through regular-expression search on the genome) was considered to be non-methylated if its inter-pulse duration ratio (ipdR) score fell below the under-methylated motif ipdR threshold, defined as (0.1^*^average motif ipdR)+(0.9^*^average non-motif ipdR), using only modifications of the same type (e.g., m6A) to compute the average non-motif ipdR. Motif and non-motif average ipdR values were computed on the central 60% of ranked ipdR scores to minimize the effect of outliers. For the palindromic motifs under analysis, motif instances were considered non-methylated if their ipdR ratios were below the under-methylated motif ipdR threshold on both strands and had at least twenty-fold SMRT sequence coverage. Hyper-methylated loci were detected as those with a number of motif instances in their upstream region larger than two standard deviations above the mean number of motif instances for all genome upstream regions (Mou et al., 2014). Highly-conserved motif instances were identified as those presenting fully aligned sequence contexts (41 bp) in all the species making up the panel of reference genomes.

### Non-supervised orthologous groups and annotated feature analysis

Protein sequences for each RefSeq identifier were parsed from the genome GenBank-format file and used to query the eggNOG database (4.5). eggNOG identifiers, categories, and descriptions were retrieved from the eggNOG database (eggNOG, RRID:SCR_002456) using HMMER (Hmmer, RRID:SCR_005305) (Eddy, 2011; Powell et al., 2014) and used to annotate extracted genome features. NOG (Non-supervised Orthologous Groups) category enrichment for a subset of methylation marks (e.g., conserved GTWW**A**C marks) in a given region relative to annotated protein coding genes (upstream, intragenic or downstream) was assessed by performing a Fisher exact test on NOG categories, using the presence of at least one such methylation mark in the region of interest as an indicator function for all genome protein coding genes with annotated NOGs. Modification mark enrichment for specific NOGs and gene-relative regions was assessed through permutation analysis, generating 10,000 NOG replicates containing the same number of genes mapping to the NOG and assessing their normalized modification density in the region under study. Modification mark enrichment for a specific annotated feature (e.g., genes with “transposase” in their product/NOG description) was assessed by performing a Mann-Whitney *U*-test on the normalized modification density of genes containing the annotated feature vs. all other genome genes, and by computing the point-biserial correlation coefficient between normalized modification densities in contiguous 1,000 bp sequence chunks and the presence of the annotated feature within said chunks. Statistical computations were performed using the Python SciPy library (SciPy, RRID:SCR_008058). When appropriate, *p*-values were adjusted for multiple hypothesis testing using the Bonferroni procedure (Dunn, 1961). Statistical significance was determined at significance level α = 0.01 for all tests reported in this work.

### Promoter analysis

Upstream regions of interest were analyzed for the presence of promoter elements using three different prediction tools: the Phi-Site Promoter Hunter (phiSITE, RRID:SCR_014754) (Klucar et al., 2010), PePPER (PePPER Prokaryote Promoter Prediction, RRID:SCR_014740) (de Jong et al., 2012) and BPROM (SoftBerry, RRID:SCR_000902). Only the strongest prediction of each method on each strand, when applicable, was considered.

## Results

### Identification of methylation motifs in *R. solanacearum*

SMRT sequencing of *R. solanacearum* GMI1000 and UY031 strains yielded different total numbers of statistically significant modification marks (229,207 for *R. solanacearum* GMI1000 and 22,732 for *R. solanacearum* UY031). These numbers correlate with a threefold difference in average sequencing coverage for detected modification marks between both strains (177.35 ± 19.46 for GMI1000 vs. 51.53 ± 23.99 for UY031) (Table 1). It is of note that most of the additional identified marks in GMI1000 correspond to m4C modifications, whereas the number of m6A modifications appears to be constant between both strains. This is consistent with lower detection yields for m4C methylation with reduced coverage (Schadt et al., 2013; Blow et al., 2016). Motif analysis of the modification profiles identified two m6A and two m4C novel methylation motifs. The two m4C motifs (C**C**CAKNAVCR and YG**C**CGGCRY) were only detected in *R. solanacearum* GMI1000, while one of the m6A methylation motifs (CAACR**A**C) was identified only in *R. solanacearum* UY031. The remaining m6A motif (GTWW**A**C) was consistently detected in both strains.

**Table 1:** **Summary statistics for modification profiles of *R. solanacearum* GMI1000 and *R. solanacearum* UY031 strains**.

**Modification type**	**Motif**	**UY031**	**GMI1000**
Not determined	–	17,989	202,350
	CAACRAC	38	0
	GTWWAC	10	1
	CCCAKNAVCR	0	358
	YGCCGGCRY	0	2,296
	All	18,094	205,005
m6A	–	373	880
	CAACRAC	2,100	0
	GTWWAC	689	779
	All	3162	1659
m4C	–	1,293	17,916
	CCCAKNAVCR	0	77
	YGCCGGCRY	0	922
	All	1,293	18,915
Expected	–	160	0
	CAACRAC	6	0
	GTWWAC	17	4
	CCCAKNAVCR	0	712
	YGCCGGCRY	0	2912
	All	183	3,628
All		22,732	229,207

*Reported numbers are for statistically significant modification marks on either DNA strand*.

### Motif-methylase assignment and distribution of predicted RM systems

Of the four detected novel motifs, only the two m6A motifs could be reliably assigned to predicted methylases in REBASE (Table 2). The CAACR**A**C motif is most likely the target of the Rso31ORF11320P fused RM system of *R. solanacearum* UY031, which has no detectable homologs in the reference panel of complete *Ralstonia* genomes. In contrast, the GTWW**A**C motif was assigned to the M.Rso31ORF22890P/M.RsoORF1982P MTase (encoded by RS_RS09960 and RSUY_RS11230, respectively, in *R. solanacearum* GMI1000 and *R. solanacearum* UY031), which is conserved in the reference panel of *Ralstonia* spp. genomes and across the *Burkholderiaceae*. Methylome analysis of *Burkholderia pseudomallei* strains had previously identified a similar type II motif (GTAW**A**C), which is likely the target of the M.Rso31ORF22890P/M.RsoORF1982P MTase homolog in *B. pseudomallei* (Nandi et al., 2015). The distribution of RM systems in both strains is similar and consistent with the overall distribution of RM systems predicted by REBASE in *Ralstonia* (Supplementary Table 4). Both strains harbor a type I RM system conserved among all *R. solanacearum* reference genomes, as well as two well-conserved type II MTases (in addition to M.Rso31ORF22890P/M.RsoORF1982P). It is worth noting that these two tightly linked MTases reside in the megaplasmid of *R. solanacearum* UY031, but are located in the chromosome of *R. solanacearum* GMI1000. Besides the fused RM system targeting the CAACR**A**C motif, *R. solanacearum* UY031 also harbors a type II RM system predicted by REBASE to target a GAN**T**C motif, although this motif was not detected by SMRT sequencing. *R. solanacearum* GMI1000 carries an additional type II RM system, as well as two type II MTases, but the C**C**CAKNAVCR and YG**C**CGGCRY motifs could not be reliably assigned to predicted methylases in this strain. In general, the RM systems and MTases not conserved between *R. solanacearum* GMI1000 and UY031 do not present homologs among other *Ralstonia* species and thus appear to have been independently acquired by each strain.

**Table 2:** **REBASE annotated methylases and associated motifs in *R. solanacearum* GMI1000 and *R. solanacearum* UY031**.

***R. solanacearum*** **UY031**				***R. solanacearum*** **GMI1000**				**RM system**				
**Replicon**	**Locus tag**	**Protein ID**	**REBASE ID**	**Replicon**	**Locus tag**	**Protein ID**	**REBASE ID**	**R/M**	**Type**	**Motif**	**% detect**.	**Cons**.
NZ_CP012687.1	RSUY_RS00445	WP_003261699.1	Rso31ORF920P	NC_003295.1	RS_RS17020	WP_011003276.1	RsoORF3394P	R	I	–	–	9
NZ_CP012687.1	RSUY_RS00435	WP_003261701.1	M.Rso31ORF920P	NC_003295.1	RS_RS17010	WP_011003274.1	M.RsoORF3394P	M	I	–	–	9
NZ_CP012688.1	RSUY_RS22775	WP_039562516.1	M1.Rso31ORF46860P	NC_003295.1	RS_RS04210	WP_011000797.1	M1.RsoORF844P	M	II	–	–	7
NZ_CP012688.1	RSUY_RS22780	WP_039562519.1	M2.Rso31ORF46860P	NC_003295.1	RS_RS04215	WP_011000798.1	M2.RsoORF844P	M	II	–	–	10
NZ_CP012687.1	RSUY_RS11230	WP_020957142.1	M.Rso31ORF22890P	NC_003295.1	RS_RS09960	WP_011001918.1	M.RsoORF1982P	M	II	GTWW**A**C	95.5/99.5	12
NZ_CP012687.1	RSUY_RS05525	WP_039558712.1	Rso31ORF11320P	–	–	–	–	RM	II	CAACR**A**C	97.0	1
NZ_CP012687.1	RSUY_RS05480	WP_003264976.1	M.Rso31ORF11220P	–	–	–	–	M	II	–	–	1
NZ_CP012687.1	RSUY_RS05495	WP_049280918.1	Rso31ORF11260P	–	–	–	–	R	II	(GAN**T**C)	–	1
NZ_CP012687.1	–	–	M.Rso31ORF11260P	–	–	–	–	M	II	(GAN**T**C)	–	1
–	–	–	–	NC_003295.1	RS_RS17175	(pseudo)	M.RsoORF3438P	M	II	(YG**C**CGGCRY)	32.8	1
–	–	–	–	NC_003295.1	RS_RS25190	WP_011003318.1	V.RsoORF3438P	V	II	–	–	1
–	–	–	–	NC_003296.1	RS_RS19865	WP_011003871.1	M.RsoORF570P	M	II	–	–	1
–	–	–	–	NC_003295.1	–	–	M.RsoORF869P	M	II	(C**C**CAKNAVCR)	17.3	4

*R denotes restriction enzymes, M methylases and V nicking enzymes associated with m5C−MTases. Bracketed motifs indicate tentative, unconfirmed mappings to RM systems. “% detect.” indicates the percentage of motif instances detected as methylated through the SMRT sequencing in either strain. “Cons.” indicates the number of species from the reference panel in which the components of the RM system are conserved, as determined through reciprocal BLAST. The “–” symbol indicates missing RM components or their missing annotation in RefSeq records. (pseudo) indicates that the corresponding locus has been annotated as a pseudogene in RefSeq*.

### Gene-relative distribution of methylation marks

**Figure 1:**
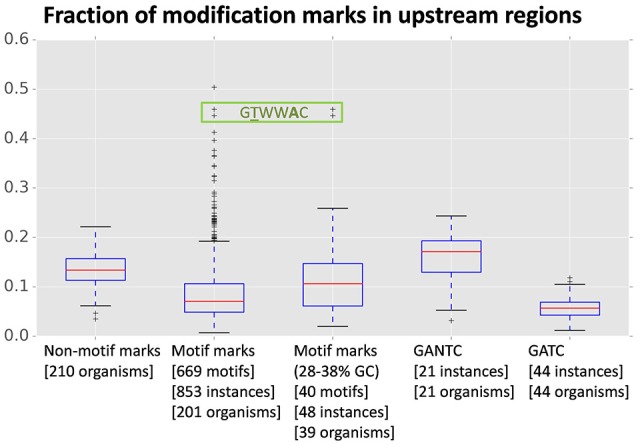
**Fraction of modification marks mapping to upstream regions of annotated genes across a panel of 210 methylomes**. The fraction of upstream marks is relative to the sum of marks mapping to upstream, downstream, and intragenic regions of annotated genes in each genome. The boxplot columns designate different datasets: non-motif associated modification marks, motif-associated modification marks, %GC-controlled (28–38% GC) motif-associated modification marks, and marks associated with the widely distributed GTN**A**C and GT**A**C motifs. For each column, the bracketed numbers in the abscissa legend indicate the number of unique motifs in the dataset, the number of instances of those motifs identified in the complete set of methylomes and the number of organisms on which such instances were detected. The data points corresponding to the *R. solanacearum* GMI1000 and *R. solanacearum* UY031 GTWW**A**C motifs are boxed.

An analysis of mark distribution with respect to annotated gene features in *R. solanacearum* strains GMI1000 and UY031 revealed that GTWW**A**C marks show a clear preference for the upstream regions of annotated genes (38% of GTWW**A**C marks vs. 8% of other motif marks) in both strains. Marks for all other identified motifs show a strong association with intragenic regions, as expected under a uniform model for methylation activity (Supplementary Image 1). The skew observed for GTWW**A**C marks cannot be explained simply by a difference in the %GC-content of the GTWW**A**C motif, since such a dramatic tendency is not observed for intergenic regions or among non-motif associated marks. To contextualize the preference of GTWW**A**C marks for upstream regions, we analyzed the distribution of modification marks with respect to annotated genes across the two *R. solanacearum* strains and a panel of 208 publicly available methylomes (Blow et al., 2016). Our results indicate that the preference of GTWW**A**C marks for upstream regions is exceptional among previously reported methylomes (Figure 1). Even though there is substantial correlation between motif %GC content and the fraction of marks mapping to upstream and downstream regions (Pearson *r* = −0.41 and *r* = −0.34, respectively; Supplementary Image 2), the preference of GTWW**A**C marks for upstream regions is distinctly high even when controlling for %GC content. Furthermore, among all the previously reported motifs showing strong (1st percentile) preference for upstream regions, only the GTWW**A**C motifs of *R. solanacearum* strains GMI1000 and UY031 show also heavy differential enrichment in upstream regions vs. downstream ones, suggesting that upstream GTWW**A**C marks may play a functional role in these *R. solanacearum* strains (Supplementary Table 5).

### Analysis of conserved methylation marks

**Figure 2:**
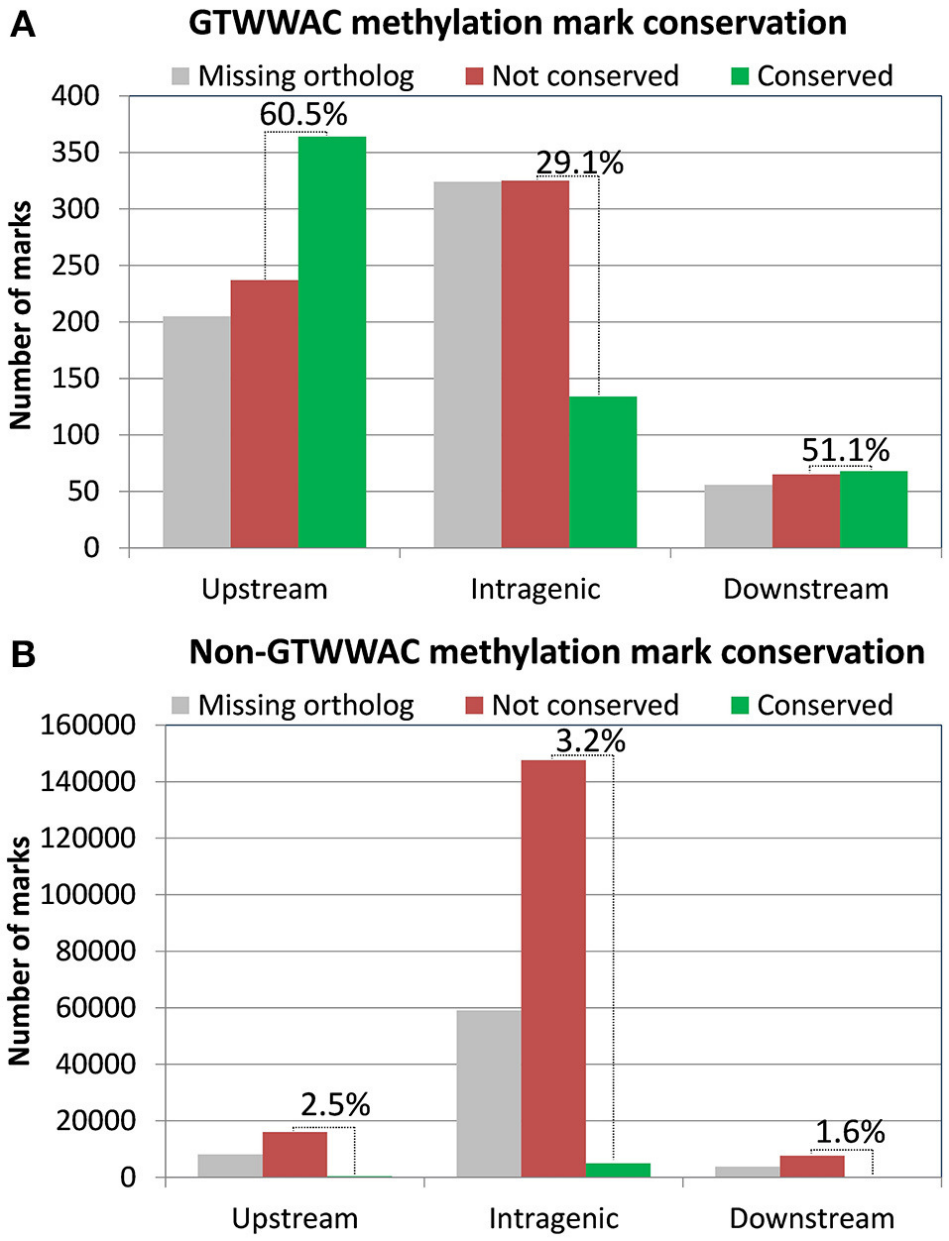
**Distribution of GTWWAC methylation marks conserved in *R. solanacearum* GMI1000 and *R. solanacearum* UY031 as a function of their location relative to annotated genes**. The plot shows the number of GTWW**A**C methylation marks conserved in each location category. Non-conserved marks are distinguished from those mapping to genes lacking an identifiable ortholog in either strain. The relative conservation of GTWW**A**C methylation marks in each region (excluding marks mapping to genes lacking orthologs) is indicated on top of the bars. **(A)** GTWWAC methylation marks. **(B)** Non-GTWWAC methylation marks.

The presence of a conserved MTase associated with a GTWW**A**C motif in both *R. solanacearum* GMI1000 and UY031 indicates that the GTWW**A**C methylome most likely predates the split between these two strains, enabling us to perform a comparative analysis of detected methylation marks associated with this motif (Supplementary Table 6). After detecting bona fide gene orthologs between both strains, we identified their conserved GTWW**A**C marks as those presenting at least 70% identity in a gapless alignment of the methylation mark sequence context (41 bp) of both strains. Analysis of mark conservation based on their location relative to annotated genes revealed that GTWW**A**C marks located upstream and downstream of annotated genes were much more likely to be conserved than those mapping to intragenic regions (Figure 2A). For marks mapping to conserved orthologs, 60.5% were conserved between both strains for upstream regions, 29.1% for intragenic regions and 51.1% for downstream regions. This association between mark location and conservation was not observed in marks not associated to the GTWW**A**C motif (Figure 2B). Among these, only intragenic regions showed a moderate amount of conservation (3.21%), most likely arising from increased sequence conservation within coding regions. The high fraction of GTWW**A**C marks mapping to upstream regions in both strains and their remarkable inter-strain conservation is hence highly suggestive of a functional role.

**Figure 3:**
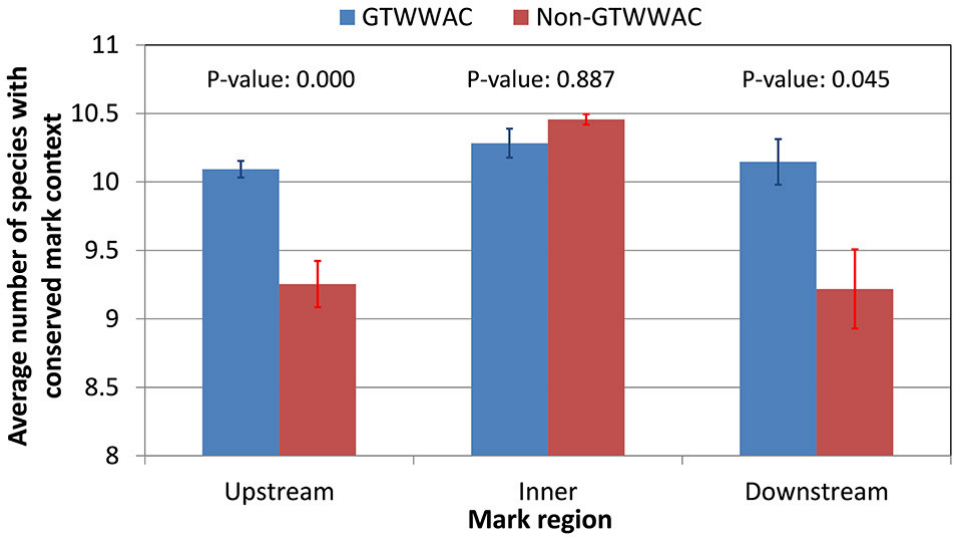
**Conservation of GTWWAC and non-GTWWAC associated marks across a panel of 12 reference complete *Ralstonia* genomes**. The plot shows the average number of genomes in which the *R. solanacearum* GMI1000 context of a methylation mark is considered to be conserved (alignment identities above 70%) for different regions (upstream, intragenic, and downstream) relative to genes with orthologs in *R. solanacearum* GMI1000 and *R. solanacearum* UY031. Vertical bars indicate the standard error of the mean. The *p*-values of a two-tailed Mann Whitney *U***-**test between GTWW**A**C and non-GTWW**A**C associated marks are provided on top of the bars.

**Figure 4:**
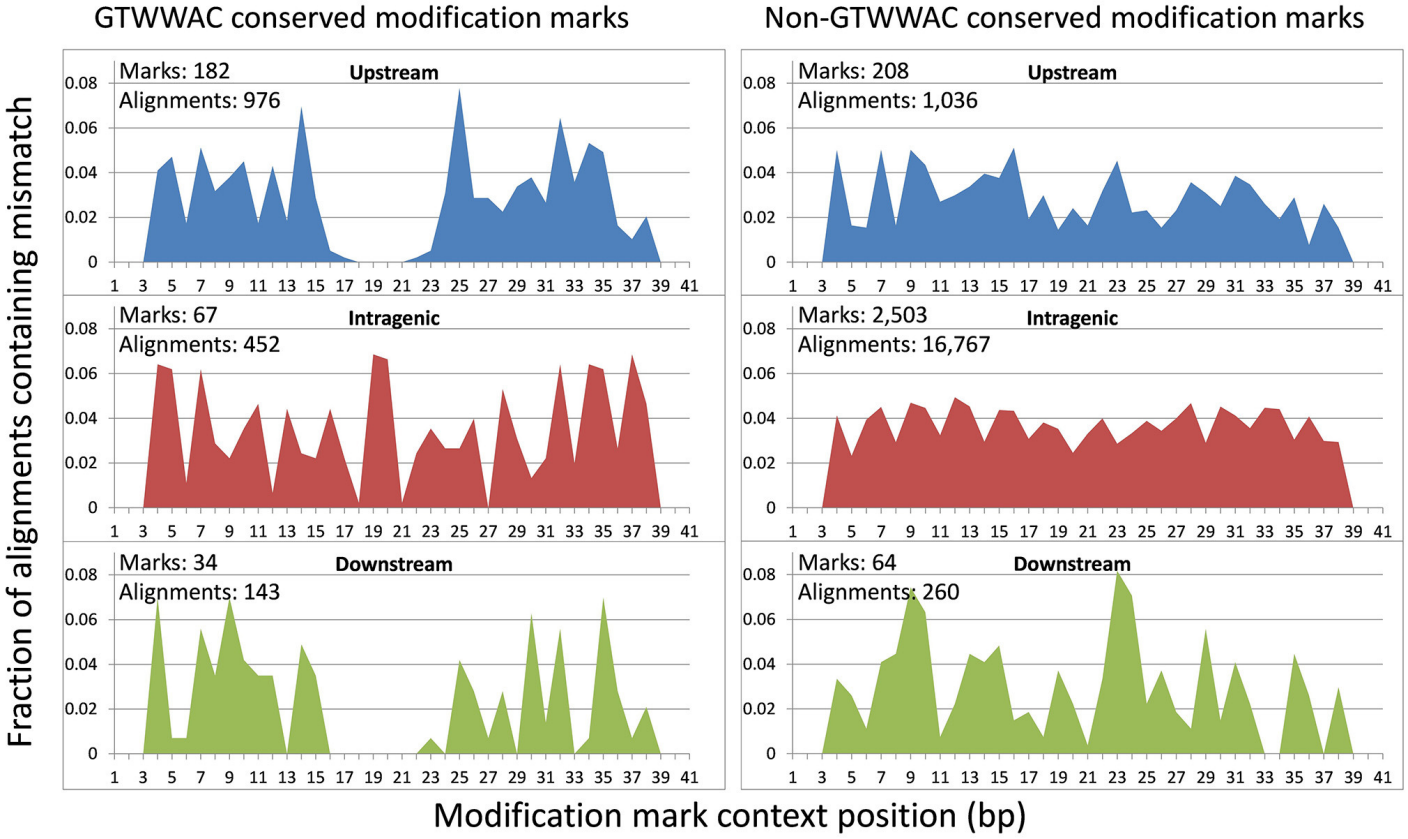
**Positional distribution of nucleotide changes with respect to *R. solanacearum* GMI1000 in gapless alignments of conserved modification marks**. The plots show the fraction of alignments containing mismatches at each alignment position for marks conserved in *R. solanacearum* GMI1000 and UY031 strains located in upstream, downstream and intragenic regions. The fraction is computed based on cumulative alignment mismatch counts for full gapless BLAST alignments (100% coverage) against a panel of 12 complete *Ralstonia* genomes. The number of conserved marks in each region, and the number of full alignments used to tally mismatches are provided. Mismatches on the first and last two positions of the alignment are not expected due to the greedy nature of the BLAST hit extension process.

To investigate the putative functional role of upstream GTWW**A**C marks, we performed a comparative analysis of conserved GTWW**A**C marks across a panel of 12 *Ralstonia* species with complete sequenced genomes, using conserved non-GTWW**A**C marks as a control. The results of this analysis were in broad agreement with those obtained in the comparison between *R. solanacearum* GMI1000 and *R. solanacearum* UY031 (Supplementary Table 6). The contexts of GTWW**A**C marks were more frequently conserved than those of non-GTWW**A**C marks in both upstream and downstream regions, although the difference is significant (Mann-Whitney U *p*-value < 0.01) only for upstream marks (Figure 3). Furthermore, among conserved mark contexts the 6 bp region corresponding to the GTWW**A**C mark is significantly well-preserved for upstream marks, but not for intragenic or downstream ones (Supplementary Image 3). Analysis of the mutational profile along fully aligned mark contexts revealed a clear pattern of sequence conservation surrounding the GTWW**A**C mark region (positions 17–22) in upstream regions (Figure 4). This pattern can also be observed in downstream regions, but is completely absent in intragenic regions and it was not observed in any region among non-GTWW**A**C conserved modification marks. This is consistent with a scenario of purifying selection acting on GTWW**A**C marks in upstream and downstream regions.

### Distribution of upstream sites in hyper-methylated and non-methylated loci

**Figure 5:**
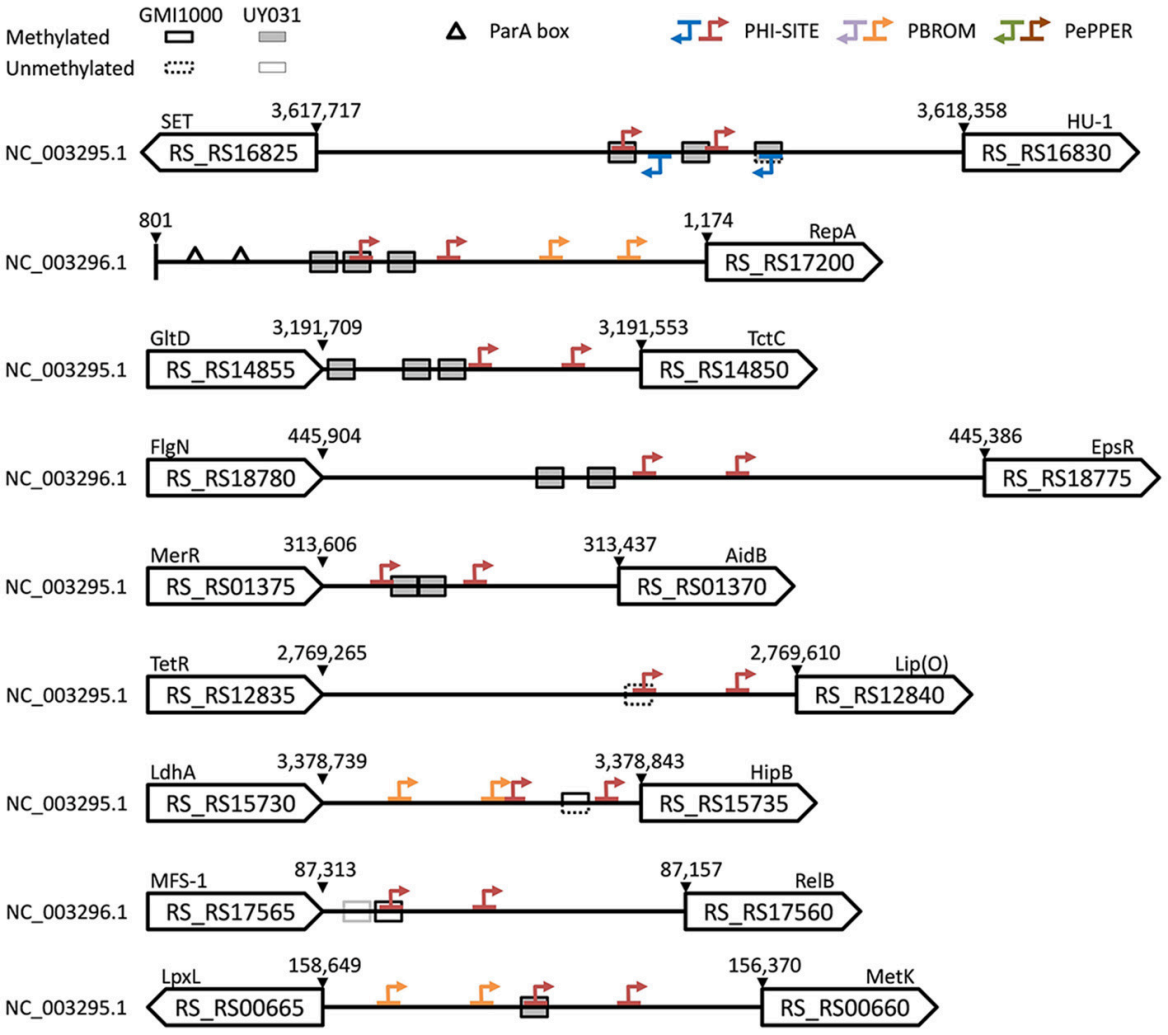
**Schematic representation of the upstream region for conserved loci enriched in methylated, non-methylated and highly-conserved GTWWAC sites**. Accessions, locus tags and coordinates are provided for the *R. solanacearum* GMI1000 genome. A mapping to old GMI1000 locus tag identifiers is provided in Supplementary Table 2. When not annotated in *R. solanacearum* GMI1000, gene acronyms are derived from homology searches against the *E. coli* genome or from representative domains (uppercase). GTWW**A**C sites are denoted by boxes, with their methylation state in *R. solanacearum* GMI1000 indicated by solid/dotted outlines and their methylation state in *R. solanacearum* UY031 indicated by white/shaded fillings. Triangles denote ParA boxes annotated in the *R. solanacearum* GMI1000 genome. Arrows indicate directional −35 and −10 promoter elements predicted by Phi-Site, BPROM, and PePPER. When predictions overlap, the results are shown using the following coloring precedence: Phi-Site, BPROM, and PePPER.

It has been proposed that the presence of upstream sites matching a methylation motif but with no apparent methylation may be indicative of an interplay between transcription factors and MTases, as evidenced by the well-studied *Escherichia coli* Pap system (Braaten et al., 1994; Low and Casadesús, 2008; Blow et al., 2016). Conversely, an overabundance of upstream methylation marks in certain loci might also be indicative of a functional role, as in the case of DNA replication control (Løbner-Olesen et al., 2003; Blow et al., 2016). To further explore the functional role of upstream GTWW**A**C sites, we identified loci with non-methylated GTWW**A**C motifs in strains GMI1000 and/or UY031, as well as upstream gene regions with an overabundance of conserved GTWW**A**C sites and with highly conserved GTWW**A**C motifs. Only five genomic loci presented more than one methylated GTWW**A**C site conserved upstream of orthologous genes in the GMI1000 and UY031 strains (Figure 5; Supplementary Table 7). These loci corresponded to the shared upstream region of RS_RS16825 (a SET domain-containing protein) and RS_RS16830 (a HU-like transcriptional regulator), the megaplasmid replication protein RepA (RS_RS17200), the tricarboxylate transporter component TctC (RS_RS14850), the AidB isovaleryl-coenzyme A dehydrogenase homolog (RS_RS01370) and the exopolysaccharide repressor EpsR (RS_RS18775). In two of these upstream regions, the conserved GTWW**A**C sites overlap (5 out of 6 positions) with the −35 boxes of predicted RNA polymerase binding sites (Figure 5). This is particularly true for the upstream region shared between the divergently transcribed RS_RS16825and RS_RS16830 genes, where GTWW**A**C sites overlap with high-confidence promoter elements in both strands. An analysis of all GTWW**A**C sites detected in upstream regions with predicted promoters revealed that more than 15% overlap predicted promoter elements (5% of them in 5 out of 6 positions), indicating that such arrangements are much more frequent than expected by chance (Supplementary Image 4). In hypermethylated upstream regions where GTWW**A**C sites do not show a clear overlap with −35 elements, they often define (RS_RS18775) or are part of larger (RS_RS01370) palindromic elements that might be targeted by transcription factors.

Most GTWW**A**C motif instances in both *R. solanacearum* GMI1000 and *R. solanacearum* UY031 were detected as methylated by SMRT sequencing. Our analysis revealed only seven upstream regions with non-methylated GTWW**A**C sites in either strain (Supplementary Table 8). Of these, only three were conserved in both strains, but they displayed different methylation states (Figure 5). The GTWW**A**C site upstream of RS_RS12840, a putative DUF3313 domain-containing lipoprotein, was non-methylated in both strains and overlapped the −35 region of a putative promoter. In contrast, the site upstream of RS_RS15735, a HipB-like transcriptional regulator, was non-methylated in strain GMI1000, but hemi-methylated in UY031. Lastly, the site upstream of RS_RS17560, a predicted RelB antitoxin, was fully methylated in GMI1000, but non-methylated in UY031. This site also overlapped a predicted −35 element and was found to be adjacent to an additional GTWW**A**C site in strain GMI1000 that is not conserved in UY031. Analysis of GTWW**A**C site conservation across the reference genome panel revealed two sites with fully aligned sequence contexts in all reference genomes (Supplementary Table 6). One of these sites mapped to the shared upstream region of the divergently transcribed *metK* (RS_RS00660) and *lpxL* (RS_RS00665) genes, where it overlaps the −35 element of a predicted *metK* promoter (Figure 5).

### NOG category enrichment analysis

To elucidate whether MTases with associated motifs preferentially target a functional subset of genes, we performed a functional category enrichment of motif-associated methylation marks based on their location (upstream, intragenic and downstream) relative to the protein-coding genes mapping to each Non-supervised Orthologous Group (NOG). Analysis of both conserved and strain-specific GTWW**A**C marks revealed no statistically significant enrichment in any NOG category. In contrast, intragenic CAACR**A**C marks showed significant enrichment for the M functional category (Cell wall/membrane/envelope biogenesis) (Supplementary Table 9). Analysis of the protein coding genes mapping to this NOG category showed that the observed enrichment was mainly driven by porins and membrane transporters, with a substantial presence of RHS repeat-containing proteins (PF05593; Pfam, RRID:SCR_004726) linked to type IV and type VI secretion systems (Koskiniemi et al., 2013). Such association could not be attributed to a simple overlap between repeat motifs and the CAACR**A**C target motif, since the codons encoding the signature motifs of RHS repeats (YD, RY and GR dipeptides) are not contained within the CAACR**A**C pattern (Hill et al., 1994).

### Genome-wide analysis of modification profiles

**Figure 6:**
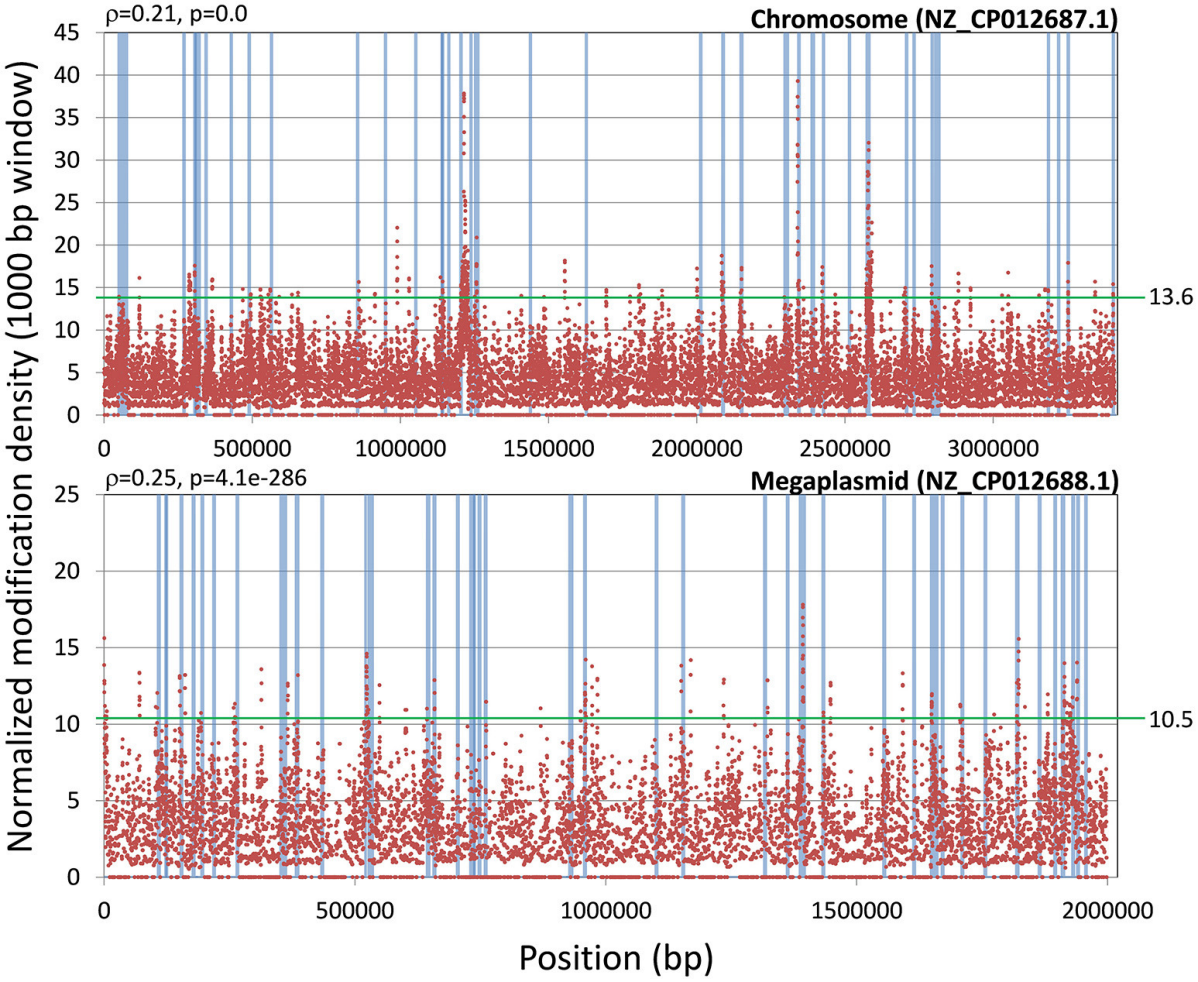
**Association of normalized modification mark density with transposable elements**. The plot shows the genomic distribution of normalized modification mark density using a 1,000 bp window with a 100 bp step size on the *R. solanacearum* UY031 chromosome and megaplasmid. The presence of transposable elements within the sliding window is indicated by light blue bars. The point-biserial correlation coefficient and its *p*-value are provided for each replicon. A green horizontal line indicates the threshold for high modification density (three standard deviations above the mean normalized modification density).

Even when restricting the analysis to modification marks with significant coverage, the fraction of modifications detected by SMRT sequencing-based analyses that can be unambiguously mapped to MTase activity remains consistently small (Schadt et al., 2013; Blow et al., 2016). As it can be seen in Table 1, in both strains the majority (99%) of these modifications correspond to unresolved modifications (i.e., SMRT sequencing was not able to assign a specific modification type (m4C or m6A)). To investigate whether these unassigned modifications might have a functional role, we first performed a comparative analysis of unassigned modification density for protein coding genes assigned to NOGs in *R. solanacearum* strains GMI1000 and UY031. We identified NOGs with unusually high unassigned modification density in their upstream, intragenic and downstream regions as those with a normalized modification density within the 5th percentile for that region in both strains. This procedure identified 27 NOGs with unusually high modification density in each of the analyzed regions (9 upstream, 15 intragenic and 3 downstream) (Supplementary Table 10), but revealed no apparent functional association among them. To further explore the possibility of a functional role for unassigned modification density, we analyzed the normalized modification density profile for the chromosome and megaplasmid of the GMI1000 and UY031 strains, computed on overlapping 1,000 bp segments. Inspection of highly-modified segments (3 standard deviations above the average modification density) revealed a consistent association between high modification density and annotated transposase genes in *R. solanacearum* UY031 (Figure 6). This association was positive and statistically significant in strain UY031 (Mann-Whitney U *p*-value < 0.01, point-biserial correlation coefficient *r* = 0.21, *p* < 0.01 (chromosome) and *r* = 0.25, *p* < 0.01 (megaplasmid), but was not detectable in GMI1000 [*r* = −0.03, *p* < 0.01 (chromosome) and *r* = −0.09, *p* < 0.01 (megaplasmid)]. A systematic analysis of publicly available methylomes (Blow et al., 2016) revealed that very few prokaryotic species show a consistent association between hyper-modification and annotated transposase genes. When detectable, this association is strongest within the intragenic and downstream regions of these genes, but this phenomenon was remarkably more pronounced in *R. solanacearum* UY031 than in any other species (Supplementary Table 11).

**Figure 7:**
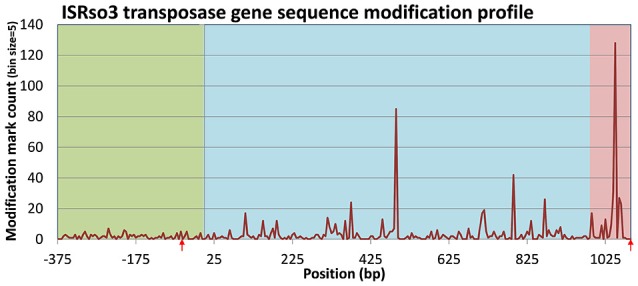
**Distribution of modification marks along the ISRso3 transposase [WP_003261205.1; ENOG4105F2I] of *R. solanacearum* UY031**. The plot shows aggregated modification mark counts in upstream, intragenic and downstream regions of the 86 genes coding for WP_003261205.1 in *R. solanacearum* UY031. Mark counts were computed on 5 bp bins. Upstream, intragenic and downstream regions are delineated by shading color. Red arrows designate the location of the inverted repeats (IR) targeted by the ISrso3 transposase.

An examination of transposase genes in the *R. solanacearum* UY031 genome showed that it contains a high copy number of transposases (86) associated with the insertion sequence ISrso3 (Jeong and Timmis, 2000). This number was much higher than that observed in other *R. solanacearum* strains and corresponded to 76% of all annotated transposase genes in the UY031 genome (Supplementary Table 12). Accordingly, a permutation analysis of normalized modification density for the NOG associated with the ISrso3 transposase (ENOG4105F2I) in strain UY031 confirmed that this NOG presented an unusually high modification density (*p*-value < 0.01) in its intragenic and downstream regions, consistent with the aforementioned association between modification density and transposase genes. A positional analysis of modification marks on the 86 copies of the ISrso3 transposase revealed a highly uneven pattern of modification in these genes, with two large modification peaks in their intragenic and downstream regions (Figure 7). Analysis of these two modification peaks revealed that they are primarily led by modification of positions 487 and 1,049. The context of these two modification loci displayed only weak sequence identity (TCNGATNNANNHNNGG), but the presence of modification marks in 85 of the 86 ISrso3 transposase genes at these positions suggested that they are the result of a systematic modification process.

## Discussion

### Distribution and possible roles of RM systems in *Ralstonia solanacearum*

Even though the nature of RM systems as primary bacterial defense mechanisms has been firmly established (Tock and Dryden, 2005), there is substantial evidence supporting many additional roles for DNA methylation in bacteria (Low and Casadesús, 2008). Moreover, the nature and scope of their impact on bacterial lifestyle and evolution has not been fully elucidated (Vasu and Nagaraja, 2013). Several studies have taken advantage of SMRT sequencing to analyze and compare the methylation profile of closely related bacteria (Budroni et al., 2011; Krebes et al., 2014; Mou et al., 2014; Nandi et al., 2015). Here, we leveraged SMRT sequencing data for two relatively distant *R. solanacearum* strains (Wicker et al., 2012) to shed light on the diversity and possible roles of DNA methylation in this agriculturally important plant pathogen. Our analysis reveals a conserved architecture of RM systems across *R. solanacearum* strains, which harbor a conserved type I RM system and three conserved type II orphan MTases. The absence of this type I RM system in other *Ralstonia* species, which contain an unrelated type I RM system annotated in REBASE, points to a major evolutionary event in the divergence of species within this genus. Divergence in type I RM systems has been shown to forestall genetic exchange and drive the evolution in *Staphylococcus aureus* strains (Lindsay, 2010) and it seems therefore plausible that a similar role may have been played by type I RM systems in the evolution of *Ralstonia* species. Beyond the presence of conserved RM elements, *R. solanacearum* strains also display a similar amount of non-conserved RM systems and orphan MTases (Supplementary Table 4), that have been presumably independently acquired by each strain. The functional role of these systems remains to be elucidated, but our analysis sheds some light onto their possible origin and function. *R. solanacearum* UY031 harbors a type II fused RM system targeting a novel m6A motif (CAACR**A**C). The gene encoding this RM system (RSUY_RS05525) is located in a prophage region identified by PHAST (PHAge Search Tool, RRID:SCR_005184) as being similar to *R. solanacearum* phiRS603, a filamentous phage of *R. solanacearum* (Zhou et al., 2011; Van et al., 2014; Guarischi-Sousa et al., 2016). The protein product of RSUY_RS05525 has no homologs among completely sequenced *R. solanacearum* genomes, but is present in the draft genomes of seven other *R. solanacearum* strains. This supports the notion that this fused RM system is phage-borne and has been recently acquired by *R. solanacearum*. Given its recent acquisition, it is unlikely that this RM system has been coopted for host-specific functions in *R. solanacearum* UY031. However, the preferential targeting of membrane-associated genes by the CAACR**A**C motif (Supplementary Table 9), including several systems known to mediate in intercellular competition (Koskiniemi et al., 2013), suggests that it could potentially play a role in strain differentiation and virulence.

### A conserved type II MTase in *Ralstonia* spp. targeting gene promoter regions

The detection and independent assignment of an identical m6A methylation motif (GTWW**A**C) to orthologous loci in *R. solanacearum* strains GMI1000 and UY031 (RS_RS09960 and RSUY_RS11230, respectively) allows us to conclusively determine the association of this methylation motif with a type II orphan MTase conserved in all completely sequenced *Ralstonia* spp. genomes. Furthermore, reciprocal BLAST analyses indicate that this MTase is conserved across the *Burkholderiaceae*, consistent with the recent identification of a similar methylation motif in *B. pseudomallei* (Nandi et al., 2015). The broad conservation of this orphan MTase across the *Burkholderiaceae* family is suggestive of a functional role for GTWW**A**C methylation. Consistent with this hypothesis, genome-wide analyses of the distribution of GTWW**A**C methylation marks relative to annotated genes in both *R. solanacearum* strains revealed a highly pronounced preference for regions upstream of annotated genes (Figure 1; Supplementary Image 1). Several lines of evidence indicate that this preference does not stem solely from the relatively low %GC content of the GTWW**A**C motif. In particular, motifs with similar %GC content do not display this bias (Figure 1), and the GTWW**A**C motif does not exhibit such a pronounced preference for intergenic or downstream regions (Supplementary Tables 4, 5). Together, these data indicate that the observed preferential targeting of upstream regions by the GTWW**A**C motif is unique among previously reported motifs. Intriguingly, the association of GTWW**A**C with upstream regions is three- and seven-fold higher than the one observed in motifs with well-established roles in gene regulation (GAN**T**C and GA**T**C, respectively; Figure 1) (Low and Casadesús, 2008; Marinus and Casadesus, 2009), suggesting a functional role for GTWW**A**C methylation in upstream regions.

The hypothesis of a functional role driving the association of the GTWW**A**C motif with upstream gene regions suggests that upstream GTWW**A**C methylation marks should also be preferentially conserved. Comparison of GTWW**A**C mark context conservation mapping to orthologous loci in *R. solanacearum* GMI1000 and UY031 revealed that it is twice more likely to be conserved in upstream regions than in intragenic regions. This trend is not observed for non-GTWW**A**C mark contexts, which tend to be more conserved in intragenic regions (Figure 2). Furthermore, analysis of conserved mark contexts across a reference panel of complete *Ralstonia* spp. genomes reveals that GTWW**A**C mark contexts are also significantly more conserved in upstream regions (Figure 3). This effect could be partly ascribed to a biased distribution of upstream GTWW**A**C marks targeting highly conserved (e.g., housekeeping) genes, but NOG category enrichment of conserved GTWW**A**C marks did not reveal such a systematic bias. Moreover, the positional distribution of mismatches across a collection of fully aligned GTWW**A**C mark context hits on reference panel genomes revealed a clear footprint of sequence conservation surrounding the GTWW**A**C motif in upstream regions (Figure 4), suggesting that conservation of upstream contexts is largely driven by purifying selection on GTWW**A**C marks. Taken together, the preferential association of the GTWW**A**C motif with upstream regions and the higher conservation of GTWW**A**C marks when mapping to upstream regions provide strong support for a functional role of GTWW**A**C methylation in gene promoter regions.

### Possible functions of GTWWAC methylation

Hyper-methylation and hypo-methylation of loci have been both put forward as possible indicators of a functional interplay between methylation and biological processes operating on the DNA sequences. For instance, attenuation of leucine-reponsive regulatory protein (Lrp) binding to hemi-methylated target sites and competition between Lrp and the Dam methylase for GA**T**C sites overlapping Lrp-binding sites is known to modulate expression of the *pap* pilin promoter, driving phase variation in *E. coli* (Braaten et al., 1994; Marinus and Casadesus, 2009). In a different context, competition for hemi-methylated GA**T**C sites between SeqA and Dam near the *E. coli* origin of replication (*oriC*) and in the promoter region of the *dnaA* gene is used to synchronize chromosomal replication with cell division (Løbner-Olesen et al., 2003; Marinus and Casadesus, 2009). Similarly, the CcrM methylase of *Caulobacter crescentus* (targeting the GANTC motif but unrelated to Dam) orchestrates the morphological differentiation of *C. crescentus* cells by modulating a transcriptional cascade involving three different regulators (DnaA, GcrA and CtrA) and occluding access to the origin of replication (Marczynski and Shapiro, 2002; Marinus and Casadesus, 2009). Although several of the precise mechanisms behind these regulatory processes involving DNA methylation remain to be fully elucidated, the presence of multiple methylation target sites in upstream regions and their hemi- or non-methylated state are shared elements in all known instances of DNA methylation interplay with cellular processes (Low and Casadesús, 2008; Marinus and Casadesus, 2009). In the context of a comparative analysis, highly-conserved methylation sites also appear as likely candidates for a functional role of DNA methylation.

Analysis of loci with conserved non- and hemi-methylated GTWW**A**C sites, loci containing multiple conserved GTWW**A**C sites in their upstream regions and loci harboring highly-conserved GTWW**A**C sites identified several genes that could potentially be regulated by the GTWW**A**C MTase (Figure 5). It is worth noting that GTWW**A**C marks overlap predicted −35 or −10 hexamers corresponding to RNA-polymerase binding sites in seven out of the nine upstream regions identified in the analysis, a fact known to play a role in modulating gene expression via Dam and CcrM methylation (Marinus and Casadesus, 2009). Among conserved non- and hemi-methylated sites, GTWW**A**C sites overlap the −35 region of a predicted lipoprotein (RS_RS12840) and a putative RelBE-like toxin-antitoxin (TA) system (RS_RS17560-RS_RS17555). This promoter region of this TA system is hemi-methylated in *R. solanacearum* GMI1000 and non-methylated in strain UY031, hinting at a differential process in DNA methylation that might be linked to cell state. Regulation of TA systems through DNA methylation has not been reported to date. If confirmed, it could provide a causative mechanism for programmed switching into the viable but non-culturable (VBNC) state that *R. solanacearum* is known to enter in certain soil conditions (Grey and Steck, 2001). In this context, the presence of a highly-conserved GTWW**A**C site overlapping the −35 element of the predicted promoter of a *metK* homolog is also intriguing. MetK synthetizes SAM, the main methyl donor in *E. coli*, and its regulation through DNA methylation could therefore define a feedback loop governing DNA methylation in *R. solanacearum*. Moreover, *E. coli metK* mutants are known to undergo filamentation (Newman et al., 1998), suggesting that *metK* regulation through DNA methylation could also be involved in cell cycle control. The possibility that GTWW**A**C methylation might be involved in cell-cycle control is substantiated by the identification of a cluster of three conserved GTWW**A**C sites overlapping a predicted −35 element upstream of the megaplasmid *repA* locus (RS_RS17200). Even though these GTWW**A**C sites do not overlap predicted ParA-binding sites, and hence seem unlikely to define a Dam/CcrM-like mechanism of replication control, they could potentially co-regulate *repA* expression and thus contribute to modulate the proper partitioning of *R. solanacearum* megaplasmids (Pinto et al., 2012).

The putative role of GTWW**A**C methylation in the regulation of broad cellular processes in *R. solanacearum* is further supported by the identification of three conserved sites in the shared upstream region of the divergently transcribed RS_RS16830 and RS_RS16825 genes. Given the large size of this intergenic region (640 bp), the precise arrangement of these GTWW**A**C sites, overlapping the −35 elements of high-confidence predicted promoters for both genes, is strongly suggestive of an interplay between GTWW**A**C methylation and transcriptional initiation at these loci. RS_RS16830 encodes a HU histone-like protein, annotated as DbhB in the *Burkholderia*. DbhB homologs are known to be involved in genome-wide DNA bending that modulates transcriptional regulation in multiple loci of *Pseudomonas aeruginosa* (Bartels et al., 2001). Furthermore, besides bending-mediated transcriptional regulation, the *E. coli* HU protein also participates in control of DNA replication through interaction with DnaA (Flashner and Gralla, 1988). In this setting, it is worth noting that the divergently transcribed RS_RS16825 encodes a predicted SET domain-containing protein-lysine N-methyltransferase. Lysine methylation of histones is known to play a key role in eukaryotic epigenetic regulation by modulating histone activity (Qian and Zhou, 2006), and a similar interaction could thus be conceivably attributed to RS_RS16825 and the DbhB histone-like protein. Lastly, DNA methylation has been shown to influence the activity of several determinants of bacterial virulence, including lipopolysaccharide synthesis (Fälker et al., 2007; Marinus and Casadesus, 2009). Our analysis revealed the presence of two conserved GTWW**A**C sites upstream of the exopolysaccharide repressor EpsR (RS_RS18775). These sites are close to (13 bp), but do not overlap the predicted −35 promoter. Interestingly, the two sites are only 6 bp apart and, together, define a perfect palindromic repeat with an AT-rich spacer, which could well be the target of a transcriptional regulator. These observations suggest that EpsR transcription might be modulated by GTWW**A**C methylation, which could represent an additional layer of control on the synthesis of exopolysaccharide-I, a major virulence determinant in *R. solanacearum* (Chapman and Kao, 1998; Schell, 2000).

### Systematic modification of multi-copy transposase genes

Regulation of transposition though DNA methylation has been experimentally described for several transposable elements (Casadesús and Low, 2006). In the well-studied Tn10 and Tn5 transposons, Dam methylation of target sites impacts transposition in two different ways (Dodson and Berg, 1989; Kleckner, 1990). On the one hand, a GA**T**C site overlapping the -10 promoter element of the transposase gene is known to activate transposase transcription when hemi-methylated. On the other hand, hemi-methylation of a second GA**T**C overlapping the transposase IR site immediately downstream of the transposase gene is also required for efficient binding of the transposase. The presumed rationale for this arrangement is to synchronize transposition with chromosome replication, thereby enhancing the transmission of transposase genes while limiting their impact on chromosome stability (Kleckner, 1990). Even though motif-associated methylation sites were not preferentially detected in transposases on either *R. solanacearum* strain, analysis of unassigned modification marks revealed a clear, genome-wide association between densely modified regions and transposase genes in strain UY031, but not in GMI1000 (Figure 6). A similar association can be identified in a few other available methylomes, but the effect is not as pronounced as in *R. solanacearum* UY031, suggesting that this is an unusual property of this particular strain. Closer inspection revealed that this association was driven primarily by the presence of a high number of ISrso3 transposases in the genome of strain UY031. Interestingly, the modification pattern on ISrso3 genes was found to be remarkably non-uniform, with two well defined peaks within both the intragenic region and the region immediately downstream of the transposase gene (Figure 7). These two peaks do not coincide with previously described targets of DNA methylation in transposases, pointing to a possible hitherto unknown mechanism of transposase regulation or to a systematic bias in the incorporation of modified bases during transposition.

### Insights from methylome analyses into *R. solanacearum* biology and evolution

Beyond its economic impact on crops around the world, *R. solanacearum* is probably best known for its ability to infect a wide variety of plant hosts, fueled by rapid adaptation, changes in its effector repertoire and phylogeographic diversification. Recent advances in sequencing technology have enabled the analysis of genome-wide DNA modification profiles in bacteria, but the biological relevance of such modifications remains largely unknown. Our identification of a conserved m6A MTase in *Ralstonia* spp. preferentially targeting gene upstream regions and the observation that its methylation sites appear to be under positive selection indicate that DNA methylation is likely playing an active role in modulating the expression of many genes, including major transcriptional regulators and several genes involved in virulence and cell-state regulation. These results support the notion that DNA methylation could act as an additional layer of control on the pathogenicity of *R. solanacearum*, paving the way for targeted experimental approaches to elucidate the nature and impact of DNA methylation on *R. solanacearum* pathogenesis and its interaction with different plant hosts. Our work also examines for the first time the possible biological role of unassigned DNA modifications. The observation that transposases from high-copy insertion sequences are systematically modified and the characterization of an active, phage-borne RM system in the highly-virulent UY031 strain indicates that DNA modification may be playing an active role in controlling horizontal transfer in *R. solanacearum*, thus influencing its evolution and phylogeographic diversification. Our findings hence indicate that DNA methylation may play an important role in the pathogenesis and adaptation of *R. solanacearum* strains to their plant hosts, and should help focus subsequent *in vitro* and *in vivo* studies aimed at determining the impact of DNA methylation in this important bacterial phytopathogen.

## Author contributions

IE, MV, AG, and SG conceived the experiment and coordinated the research. LL and CV performed SMRT sequencing and motif analysis on *R. solanacearum* GMI1000. RG and JS performed SMRT sequencing and motif analysis on *R. solanacearum* UY031. IE and MP performed the comparative analyses. IE wrote the necessary scripts and performed the statistical analyses. IE, MP, MV, AG, and SG discussed the findings and interpreted the results. IE drafted the manuscript. All authors read and approved the manuscript.

## Funding

This work was funded by the Spanish Ministry of Economy and Competitiveness projects AGL2013-46898-R and AGL2016-78002-R to MV and by a U.S. National Science Foundation (MCB-1158056) award to IE. We also acknowledge financial support from the CERCA Program of the Catalan Government (Generalitat de Catalunya), the University of Maryland, Baltimore County Office of Research, the “Severo Ochoa Program for Centers of Excellence in R&D” 2016–2019 (SEV-2015-0533) of the Spanish Ministry of Economy and Competitiveness and the COST Action SUSTAIN (FA1208) from the European Union. RG is the recipient of a doctoral fellowship [grant 2012/15197-1, São Paulo Research Foundation (FAPESP)]. JS has a researcher fellowship from CNPq (304881/2015-5). MP holds an APIF doctoral fellowship from Universitat de Barcelona. This work was also performed in collaboration with the GeT core facility, Toulouse, France (http://get.genotoul.fr), and was supported by France Génomique National infrastructure, funded as part of “Investissement d'avenir” program managed by Agence Nationale pour la Recherche (contract ANR-10-INBS-09).

### Conflict of interest statement

The authors declare that the research was conducted in the absence of any commercial or financial relationships that could be construed as a potential conflict of interest. The reviewer AZ and handling Editor declared their shared affiliation, and the handling Editor states that the process nevertheless met the standards of a fair and objective review.
