# Supplementary material for: Comparative Analysis of Ralstonia solanacearum Methylomes
Source: Front Plant Sci. 2017 Apr 13;8:504. doi: 10.3389/fpls.2017.00504 (PMC5390034; doi:10.3389/fpls.2017.00504)
Supplement: Supplementary file 13 [file Image1.PDF]

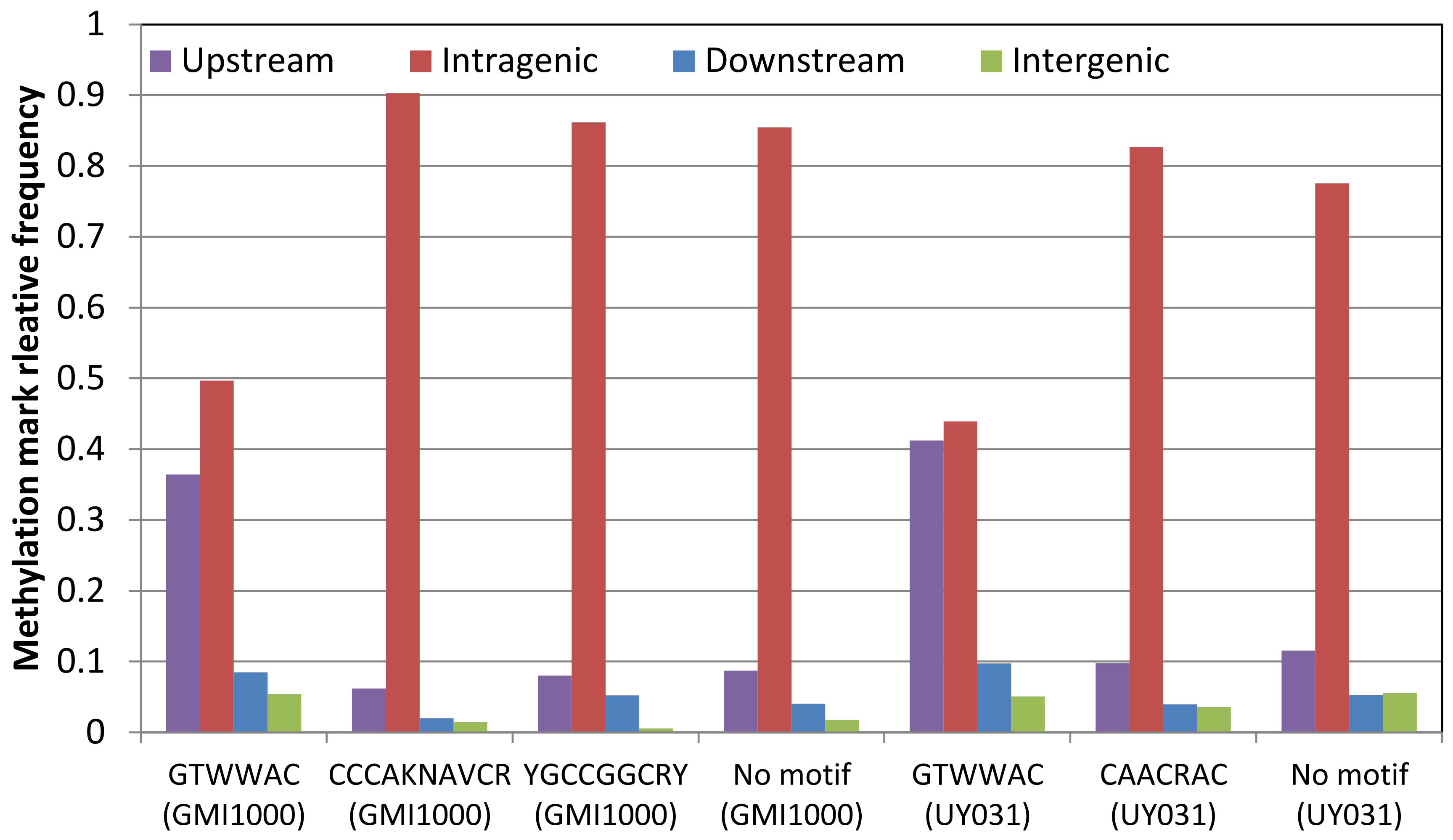

Relative frequency of motif and non-motif associated modification marks detected through SMRT sequencing as a function of their position relative to annotated genes.
