# Supplementary material for: Comparative Analysis of Ralstonia solanacearum Methylomes
Source: Front Plant Sci. 2017 Apr 13;8:504. doi: 10.3389/fpls.2017.00504 (PMC5390034; doi:10.3389/fpls.2017.00504)
Supplement: Supplementary file 14 [file Image2.PDF]

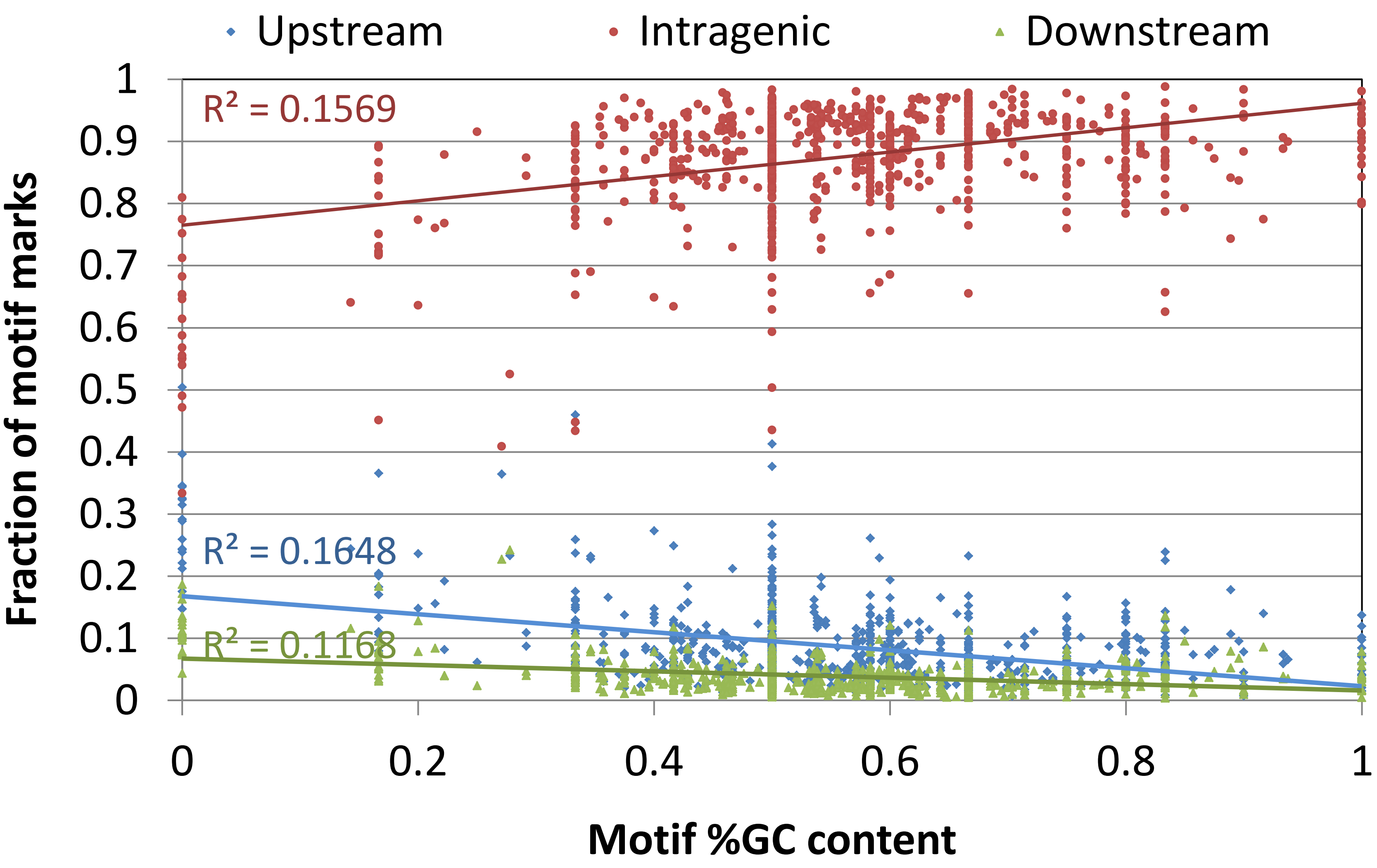

Fraction of motif marks mapping to upstream, intragenic and downstream regions of annotated genes in the methylomes of 210 bacteria, as a function of motif %GC content. The Pearson correlation coefficient (R^2) between both variables is provided for each gene-relative region. Pearson R values: $R_{upstream}$=-0.405, $R_{downstream}$=-0.341, $R_{intragenic}$=0.396.
