# Supplementary material for: Comparative Analysis of Ralstonia solanacearum Methylomes
Source: Front Plant Sci. 2017 Apr 13;8:504. doi: 10.3389/fpls.2017.00504 (PMC5390034; doi:10.3389/fpls.2017.00504)
Supplement: Supplementary file 15 [file Image3.PDF]

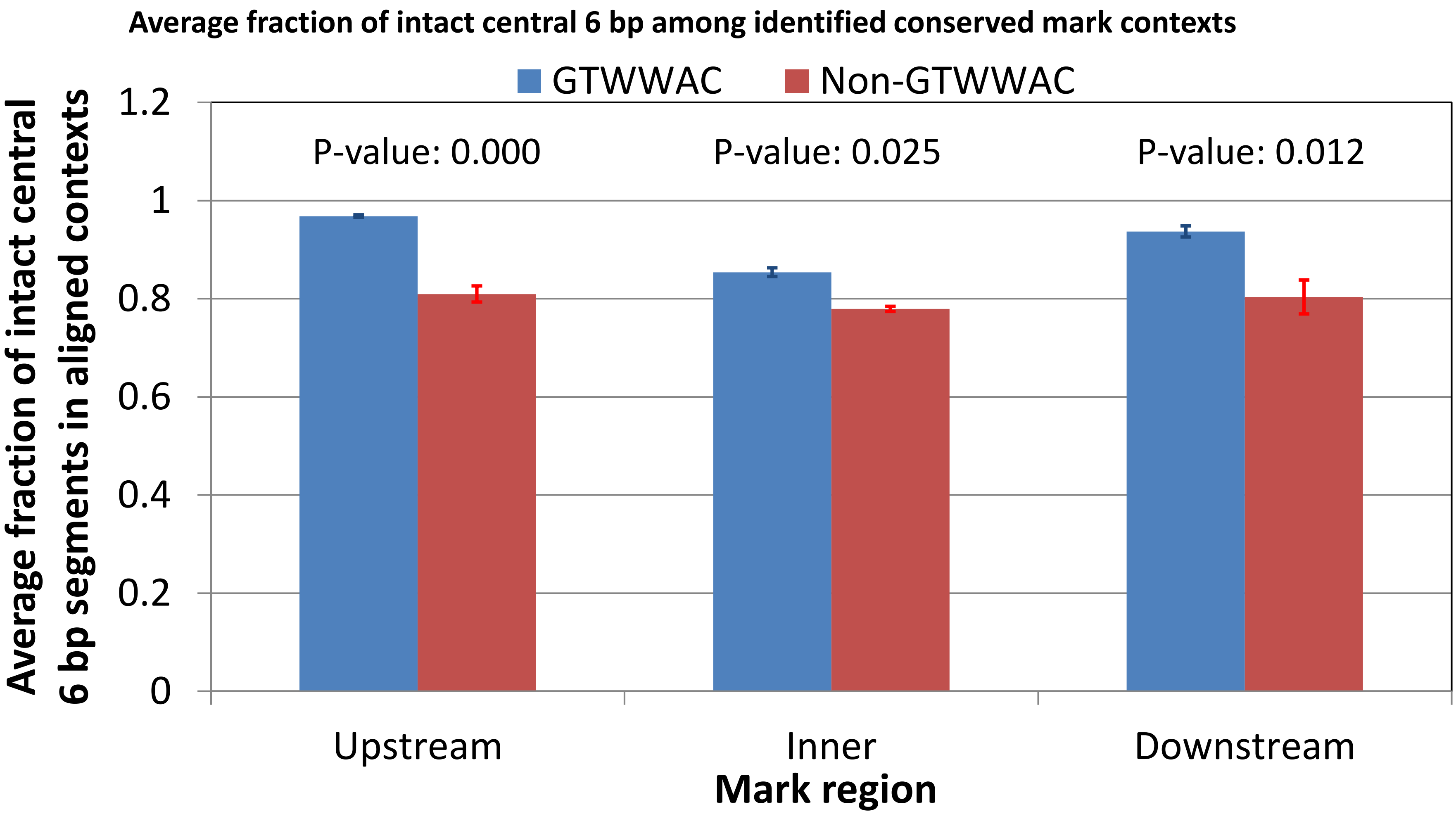

Conservation of the central 6 bp segment corresponding to the GTWW**A**C motif in alignments of GTWW**A**C and non-GTWW**A**C associated marks against a panel of 12 reference complete *Ralstonia* genomes. The plot shows the average fraction of genomes (relative to the total number of genomes in which a hit is identified) in which the central 6 bp segment of the methylation mark is fully conserved (with respect to the *R. solanacearum* GMI1000 context sequence) for different regions (upstream, intragenic and downstream) relative to genes with orthologs in *R. solanacearum* GMI1000 and *R. solanacearum* UY031. Vertical bars indicate the standard error of the mean. The p-values of a two-tailed Mann Whitney U test between GTWW**A**C and non-GTWW**A**C associated marks are provided on top of the bars.
