# Supplementary material for: Comparative Analysis of Ralstonia solanacearum Methylomes
Source: Front Plant Sci. 2017 Apr 13;8:504. doi: 10.3389/fpls.2017.00504 (PMC5390034; doi:10.3389/fpls.2017.00504)
Supplement: Supplementary file 16 [file Image4.PDF]

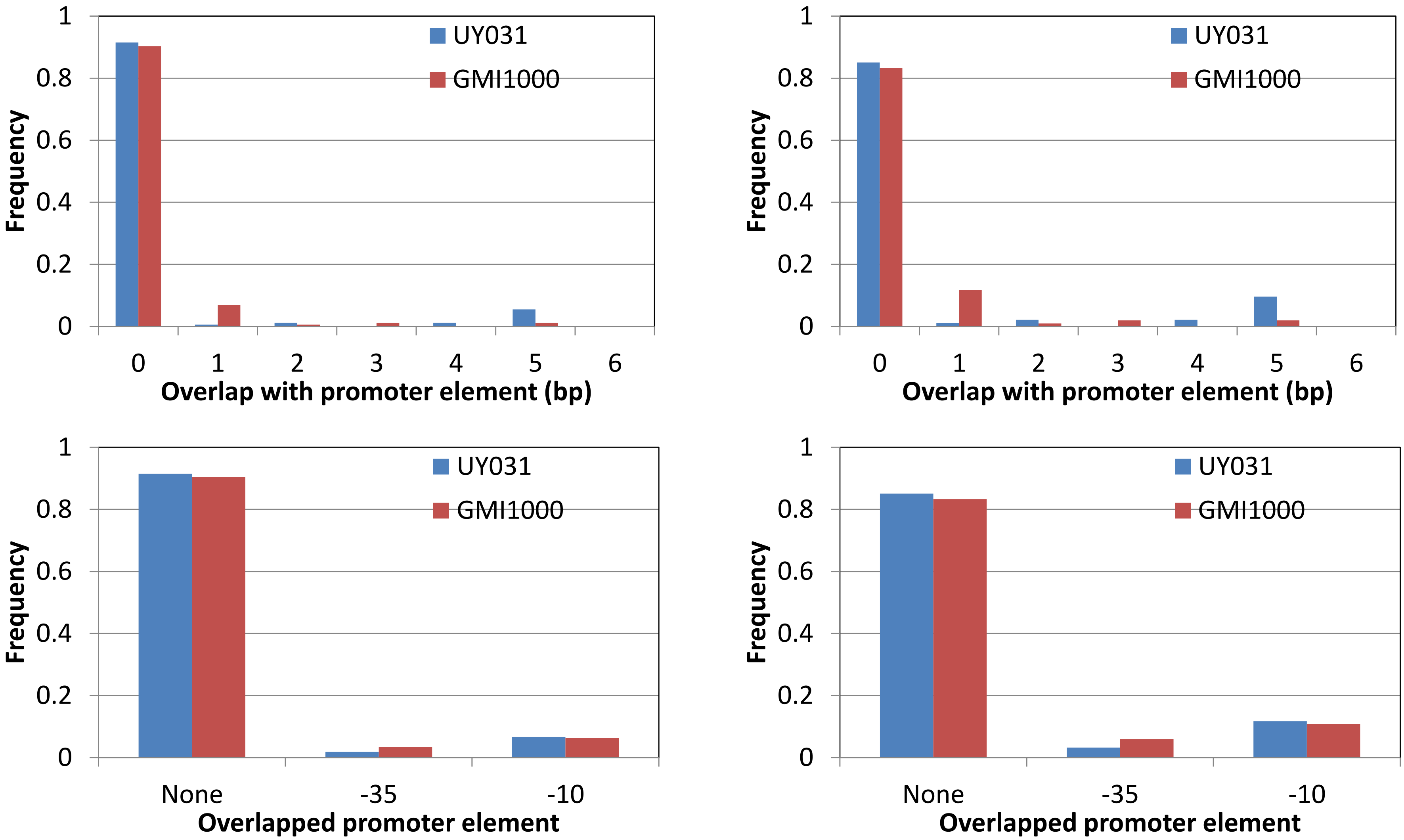

Distribution of overlaps between detected GTWWAC sites and promoters predicted using BPROM on all upstream sequences (>99 bp) containing GTWWAC sites. The plots show the distribution with respect to the amount of overlap (bp) and the type of element being overlapped (-35 or -10 region) when considering all sequences (left) or just those containing predicted promoters (right).
